# Adherence measurement considerations for oral antiretroviral medications

**DOI:** 10.1002/jia2.26397

**Published:** 2024-11-26

**Authors:** Corwin Coppinger, Peter L. Anderson

**Affiliations:** ^1^ Department of Pharmaceutical Sciences, Skaggs School of Pharmacy and Pharmaceutical Sciences University of Colorado Anschutz Medical Campus Aurora Colorado USA

1

Non‐adherence has been, and remains, the most powerful predictor of unfavourable outcomes for both pre‐exposure prophylaxis (PrEP) and anti‐retroviral therapy. In particular, non‐adherence during oral PrEP trials complicated the picture of efficacy, a fact that is again on display following the PURPOSE‐1 trial that included daily oral emtricitabine/tenofovir alafenamide (F‐TAF) and emtricitabine/tenofovir disoproxil fumarate [1]. Adherence in early trials was initially measured via self‐report, pill counts and medication dispensation records, all suggesting high adherence (>90%) during trial periods [2, 3]. However, analysis of biological samples from the active arms indicated much lower PrEP use (unquantifiable or low drug concentrations), and particularly low use among participants who seroconverted. The lessons learned from these trials include: objective adherence measures greatly outperformed indirect measures, and many participants, by virtue of repeated undetectable drug concentrations between study visits, likely had little intention of being adherent, and this was unbeknownst to study personnel. Intentionality of non‐adherence is difficult to determine and nuanced and likely underappreciated when considering adherence measurements in trials.

In this Viewpoint, we highlight the need for more research to consider adherence measurements when participants have little intention of adhering to the medication. We recognize the only way to differentiate the intentionality of non‐adherence is participant self‐report, which is subject to social desirability bias [4]. We also recognize and acknowledge that open communication is critical to this subject and appreciate that the foundation of care requires open and non‐judgemental conversations between clients and clinicians. The reasons for participating in trials are complex: for some clients, there may be an initial intent to take the medication, but subsequent side effects or perception of not being at risk anymore may temper their enthusiasm to continue to take the medication; both the incentives of trial participation (financial and access to healthcare) and/or social desirability bias (not wanting to disappoint the study staff) may lead to misreporting of non‐adherence. Nevertheless, clinicians and researchers need reliable adherence measurements to properly interpret expected/observed therapeutic outcomes, whether the participant is intentionally or unintentionally non‐adherent. Additionally, intent to adhere is important for policymakers who must target appropriate populations for medical interventions (i.e. those who intend to be adherent).

The main adherence assessment in the recently published PURPOSE‐1 study was intraerythrocytic tenofovir‐diphosphate (TFV‐DP), the phosphorylated anabolite of TFV, which is formed and trapped in red blood cells with a 17‐day half‐life. It accumulates with repeated dosing (i.e. adherence) and provides an estimate of cumulative adherence in the preceding 1–2 months, often reported as doses/week on average: <350 fmol/punch, fewer than 2 tablets per week; 350–699 fmol/punch, 2–3 tablets per week; 700–1249 fmol/punch, 4–6 tablets per week, ≥1250 fmol/punch 7 tablets per week [5]. The measurement confirms medication ingestions, is not manipulable, and detects intentional and unintentional non‐adherence alike. It is not susceptible to white‐coat dosing (being non‐adherent but dosing just prior to a clinic visit) because the drug concentration requires many days of repeated dosing to reach high concentrations—a white‐coat dose will have a negligible effect on the concentration. In fact, this type of biomarker can be combined with short half‐life drug concentrations to identify white‐coat dosing. An example was observed in the HPTN 084 study, which collected plasma TFV (a short half‐life drug concentration) along with TFV‐DP in dried blood spots (DBS; long half‐life biomarker). Twenty‐five percent of those who seroconverted exhibited high plasma TFV but very low TFV‐DP in DBS, reflecting poor cumulative adherence but dosing just before their clinic visit (i.e. white‐coat dosing). Detectable emtricitabine‐triphosphate (short half‐life) in the same DBS sample as low TFV‐DP provides the same white‐coat interpretation [6]. The limitations of intraerythrocytic TFV‐DP are pharmacokinetic variability and an inability to discern patterns of cumulative dosing. Given interindividual variability, some overlap between adjacent adherence categories is expected, although the overlap between high and low adherence is not. Drug concentrations in hair—another long half‐life biomarker—provide analogous interpretations [5]. These measurements are well‐suited for quantifying the degree of non‐adherence in all settings because they inform cumulative and recent dosing information. The PURPOSE‐1 study, where some intentional non‐adherence was possible, reported granular adherence data based on TFV‐DP in DBS, as well as an adherence‐efficacy relationship for F‐TAF [1]. These data inform biological efficacy for F‐TAF, as well as the low likelihood of daily oral PrEP uptake in this population.

Many excellent adherence reviews have been published that discuss the merits and drawbacks of other adherence assessments in the PrEP era, including self‐report, announced and unannounced pill counts, electronic diaries, electronic medication containers, digital pills and urine/plasma drug concentrations [7, 8, 9]. All have strengths and limitations and potential roles dependent on the study or clinical setting (Table 1). We wish to highlight two considerations needing more research relating to non‐adherence when participants have little intention of being adherent. The first involves requiring participant cooperation for the adherence assessment, such as electronic medication containers and digital pills. These methods rely on participants to take part in the adherence assessment—for example, participants are aware that opening the electronic medication container or ingesting the digital pill emits a signal for adherence. If participants are intentionally non‐adherent, they may refuse to participate in the adherence assessment, which threatens its utility [4]. This leads to the question: How does intentional non‐adherence influence the decision to cooperate with adherence measurements? And does the requirement to cooperate with the adherence assessment influence the individual's willingness to participate in the study?

**Table 1:** Main pros and cons of adherence measurements as they relate to non‐adherence [6, 8, 10, 11, 12, 13, 14]

Adherence measure	Objective or subjective	Pro	Con
Urine drug screening	Objective	Confirms ingestion; potential for rapid immunoassay at point of care	Only detects if dose was ingested in previous 24–48 hours; white‐coat dosing
Plasma drug concentration	Objective	Confirms ingestion; sensitive assay can detect dose from 7 to 14 days in past	Only detects most recent dose; white‐coat dosing
Dried blood spot testing	Objective	Agnostic to intentional versus unintentional non‐adherence; confirms cumulative ingestions; well‐studied	Specific to tenofovir; some overlap in adjacent adherence categories; cannot discern non‐adherence patterns
Hair testing	Objective	Agnostic to intentional versus unintentional non‐adherence; confirms cumulative ingestions; potential to test any drug; potential to assess drug along hair shaft for adherence patterns	Less pharmacokinetics information to guide interpretation; some overlap in adjacent categories; need to donate hair
Digital pills	Objective	Confirms ingestion; captures dosing patterns; high accuracy	Requires participant cooperation; potential malfunction
Electronic monitors	Objective	Captures dosing patterns	Requires some participant cooperation; potential malfunction; does not confirm ingestion
Directly observed/video observed	Objective	Captures dosing patterns	Requires participant cooperation; intrusive; may not confirm ingestion (e.g. cheeking dose)
Self‐reporting	Indirect	Convenient; inexpensive	Social desirability bias; recall limitations
Pill count	Indirect	Convenient; inexpensive; unannounced counts more reliable	Susceptible to manipulation particularly for announced counts; unannounced counts intrusive; does not confirm ingestion
Medication possession ratio	Indirect	Convenient; inexpensive	Does not confirm ingestion; requires accurate refill data

The second question relates to manipulating the adherence assessment for which there are several examples. White‐coat dosing (described above) is an example of a participant chronically missing their medication, but taking a dose just before the clinic visit, a profile observed in 25–30% of PrEP participants in some trials [6, 15]. Urine and plasma drug concentration testing (short half‐life biomarkers) are susceptible to this manipulation. Another is altering self‐reports and pill counts, which was frequently observed in early PrEP trials [2]. For example, participants may dump medication prior to clinic visits to appear adherent. This leads to the question: What is the relationship (if any) between intentional non‐adherence and potential manipulation of adherence assessments?

We believe these questions, and the need to consider intentional non‐adherence more globally, are important for future adherence measurement studies. We believe that intentional non‐adherence was common in the PrEP trials described above, but the true prevalence and clinical impact requires additional research. These issues are important, because only with robust adherence measurements can appropriate target populations be identified, and accurate estimates of drug safety and efficacy be established to guide clinical management.

## COMPETING INTERESTS

CC has no competing interests; PLA receives research contracts from Gilead, paid to his institution, outside the present work.

## AUTHORS’ CONTRIBUTIONS

Both authors researched and wrote the manuscript, and read and approved the final manuscript.

## FUNDING

The support for this research was granted by NIH RO1AI170298 (Peter L. Anderson).
